# Effects of Long Noncoding RNA H19 Polymorphisms on Urothelial Cell Carcinoma Development

**DOI:** 10.3390/ijerph16081322

**Published:** 2019-04-12

**Authors:** Po-Jen Yang, Ming-Ju Hsieh, Tung-Wei Hung, Shian-Shiang Wang, Shiuan-Chih Chen, Meng-Chih Lee, Shun-Fa Yang, Ying-Erh Chou

**Affiliations:** 1 Institute of Medicine, Chung Shan Medical University, Taichung 402, Taiwan; cshy1030@csh.org.tw (P.-J.Y.); 170780@cch.org.tw (M.-J.H.); a6152000@ms34.hinet.net (T.-W.H.); sswdoc@vghtc.gov.tw (S.-S.W.); sccy399@yahoo.com.tw (S.-C.C.); mcl@csmu.edu.tw (M.-C.L.); ysf@csmu.edu.tw (S.-F.Y.); 2 School of Medicine, Chung Shan Medical University, Taichung 402, Taiwan; 3 Department of Family and Community Medicine, Chung Shan Medical University Hospital, Taichung 402, Taiwan; 4 Cancer Research Center, Changhua Christian Hospital, Changhua 500, Taiwan; 5 Graduate Institute of Biomedical Sciences, China Medical University, Taichung 404, Taiwan; 6 Division of Nephrology, Department of Medicine, Chung Shan Medical University Hospital, Taichung 402, Taiwan; 7 Division of Urology, Department of Surgery, Taichung Veterans General Hospital, Taichung 407, Taiwan; 8 Department of Family Medicine, Taichung Hospital, Ministry of Health and Welfare, Taichung 403, Taiwan; 9 Department of Medical Research, Chung Shan Medical University Hospital, Taichung 402, Taiwan

**Keywords:** urothelial cell carcinoma, polymorphism, H19, lncRNA

## Abstract

Urothelial cell carcinoma (UCC) is one of the major malignancies of the genitourinary tract, and it is induced by carcinogenic epidemiological risk factors. H19 is one of the most crucial long noncoding RNAs (lncRNAs) and is involved in various types of bladder cancer. In this study, we examined H19 single-nucleotide polymorphisms (SNPs) to investigate UCC susceptibility and clinicopathological characteristics. Using real-time polymerase chain reaction, we analyzed five SNPs of H19 in 431 UCC patients and 431 controls without cancer. The results showed that patients with UCC carrying the H19 rs217727 CT + TT and rs2107425 CT + TT genetic variants had a high risk of developing muscle invasive tumors (pT2–T4) (*p* = 0.030; *p* = 0.025, respectively). With a median follow up of 39 months, CT+TT polymorphisms of rs2107425 were associated with worse disease-specific survival (adjusted hard ratio (AHR) = 2.043, 95% confidence interval (CI) = 1.029-4.059) in UCC patients aged older than 65 years. In conclusion, our results indicate that patients with UCC carrying the H19 rs217727 CT + TT and rs2107425 CT + TT genetic variants have a high risk of developing muscle invasive tumors. Thus, H19 polymorphisms may be applied as a marker or therapeutic target in UCC treatment.

## 1. Introduction

Bladder cancer is the fourth most common cancer in men and the eleventh most common cancer in women worldwide [1]. Approximately 90% of all bladder cancers are urothelial cell carcinomas (UCCs) [2]. In Taiwan, UCC ranks twelfth and thirteenth for mortality rates among all cancer deaths in male and female patients with bladder cancer [2,3]. Epidemiology and risk factors such as smoking, occupational exposure to polycyclic aromatic hydrocarbons, aromatic amines, and heavy metals (such as cadmium, chromium, nickel, lead), and genetic predisposition have been suggested to be associated with urothelial bladder cancer (UBC) [4,5]. Accumulating evidence suggests that long noncoding RNAs (lncRNAs) and polymorphisms in genes might play crucial roles in bladder cancer carcinogenesis and metastasis [6,7,8,9].

H19 is one of the most important lncRNAs. It is located on chromosome 11p15.5 in a cluster with insulin-like growth factor 2 (IGF2) [10]. LncRNAs are the largest family of noncoding transcripts and play crucial roles in differentiation, development, growth, chromatin dynamics, and gene expression [10]. The H19 gene is strongly expressed in many tissues from the early stages of enbryogenesis throughout fetal life [7]. It is downregulated postnatally and reactivated in various types of bladder cancer [7,11,12,13,14,15,16,17], suggesting that it plays a role as a tumor marker in bladder carcinoma [7,9,13,15]. Studies have explored the possible role of H19 single-nucleotide polymorphisms (SNPs) in various cancers, such as breast cancer [18,19,20], oral squamous cell carcinoma [21], lung cancer [22,23], hepatocellular cancer [24], and bladder cancer [6,7,25]. However, inconsistent results have been obtained regarding H19 SNPs in different cancers, and the role of H19 SNPs in carcinogenesis and metastasis remained controversial.

Although the lncRNA H19 has been implicated in the regulation of carcinogenesis and progression of various cancers, the exact role of H19 SNPs in UCC in the Taiwanese population remains unclear. In this study, we investigated five H19 SNPs, namely rs217727, rs2107425, rs2839698, rs3024270, and rs3741219, to elucidate their correlations to UCC development and clinical pathological status

## 2. Materials and Methods

### 2.1. Study Population

In this study, we enrolled 431 UCC patients between 2010 and 2015 at Taichung Veterans General Hospital in Taichung, Taiwan. Participants included 272 men and 159 women with a mean age of 68.6 ± 11.8 years. All patients had pathology-proved UCC of the upper urinary tract or bladder. Patients with UCC were clinically diagnosed and staged according to the tumor–node–metastasis staging system of the American Joint Committee on Cancer [26]. The treatment protocol of these patients included radical nephroureterectomy and bladder cuff excision with lymph node dissection, as described previously [27]. For the control group, 431 sex-matched healthy controls (272 men and 159 women with a mean age of 59.4 ± 7.1 years) who visited the hospital for physical examination were recruited for this study. Individuals in the control group had no self-reported history of cancer at any site. Information on the personal characteristics and demographic characteristics of the study participants were obtained through interviewer-administered questionnaires. Before the initiation of the study, informed written consent was obtained from all individuals enrolled. This study was approved by the Institutional Review Board of Taichung Veterans General Hospital (IRB No. CF11094).

### 2.2. DNA Extraction and H19 Genotyping

To acquire DNA for polymerase chain reactions (PCRs), DNA was extracted from EDTA-anticoagulated venous blood collected from patients with UCC and controls by using a QIAamp DNA Blood Mini Kit (Qiagen, Valencia, CA, USA) according to the manufacturer’s instructions. The extracted DNA was dissolved in Tris ethylene buffer (10 mmol/L Tris and 1 mmol/L EDTA; pH 7.8). The eluted DNA was quantified at OD260 nm on NanoDrop™ 2000 spectrophotometers. The extracted DNA was refrigerated and stored at −20 °C and was applied as a template in PCRs. Allelic discrimination assessment for H19 rs217727 (assay IDs: C___2603707_10), rs2107425 (assay IDs: AN7DPPP), rs2839698 (assay IDs: C___2603701_10), rs3024270 (assay IDs: C__15833426_10), and rs3741219 (assay IDs: C__27492510_10) was performed with a TaqMan assay by using an ABI StepOnePlus™ Software v2.3 real-time PCR system. The data acquired from real-time PCR were further evaluated using SDS 7000 series software (Applied Biosystems, Foster City, CA, USA).

### 2.3. Statistical Analysis

To compare age, sex differences, and demographic characteristic distributions between the study and control groups, Fisher’s exact test and the Mann–Whitney U-test were applied for statistical analysis. Multiple logistic regression models were used to estimate the odds ratio (OR) and 95% confidence interval (CI) of the association between genotype frequencies and UCC risk and clinical pathological characteristics; *p* < 0.05 was considered statistically significant. The experimental data were analyzed using SAS statistical software (Version 9.1, 2005; SAS Institute, Cary, NC, USA).

## 3. Results

The distribution of demographical characteristics is presented in Table 1. Analysis of study participants’ demographic characteristics revealed that 36.2% (156/431) of controls and 30.4% (131/431) of patients with UCC were smokers. Statistically significant distributional differences were observed in age (*p* < 0.001) between controls and UCC patients. However, no significant differences were observed in tobacco consumption between these two groups (*p* = 0.071).

The genotype distributions of H19 gene polymorphisms in 431 controls and 431 patients with UCC are presented in Table 2. The highest distribution was found for the H19 rs217727 and rs3741219 genetic polymorphisms in controls homozygous for CC or heterozygous for GA and UCC patients heterozygous for CT or homozygous for AA, respectively. For the H19 rs2107425, rs2839698, and rs3024270 polymorphisms, the highest distribution was found in controls and UCC patients heterozygous for CT, homozygous for CC, or heterozygous for GC, respectively. After adjustment for several variables including age, sex, and tobacco consumption, no significant differences were observed between UCC patients carrying the rs217727, rs2107425, rs2839698, rs3024270, and rs3741219 polymorphisms of the H19 gene and those carrying the wild-type (WT) gene.

To clarify the role of H19 genetic polymorphisms in UCC development, the distribution frequency of clinical statuses such as clinical stage, tumor size, lymph node metastasis, distant metastasis, and histopathological grading in UCC patients carrying H19 genetic polymorphisms was estimated. UCC patients carrying the H19 rs217727 and rs2107425 CT + TT genetic variants had a higher risk of muscle invasive tumors (OR = 1.534, 95% CI = 1.042–2.258, *p* = 0.030; OR = 1.586, 95% CI = 1.06–2.373, *p* = 0.025, respectively) than those carrying the WT gene did, but no significant differences were observed in tumor T status, lymph node status, metastasis, or histopathological grading (Table 3 and Table 4).

**Figure 1:**
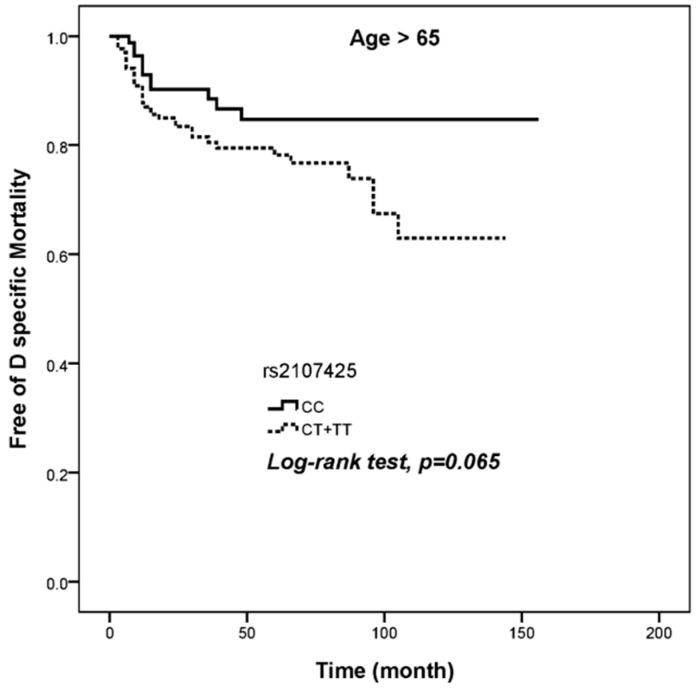
Analysis of H19 rs2107425 polymorphism and survival in UCC patients aged older than 65 years. Disease specific mortality among the two phenotypes of polymorphisms at H19 rs2107425 in patients aged older than 65 years.

Regarding the risk of disease-specific mortality in 264 patients aged older than 65 years (Table 5), patients with a CT+TT alleles at rs2107425 had a higher risk of disease-specific mortality (AHR = 2.043, 95% CI = 1.029–4.059), but no significant differences were observed in rs217727, rs2839698, rs3024270, and rs3741219 polymorphisms of the H19 gene. Furthermore, as shown in Figure 1, patients with the CT+TT genotype at rs2107425 showed a trend towards having a poor prognosis and lower disease-specific survival as compared with the WT (log rank test, *p* = 0.065).

## 4. Discussion

In this study, we revealed the association of H19 SNPs with UCC susceptibility and clinical status. Smoking is the most common and well-known risk factor, and it accounts for approximately 50% of UBC cases [4]. Furthermore, cigarette smoking is associated with promoter DNA hypermethylation in bladder cancer tumor suppressor genes [28,29]. DNA methylation in tumor suppressor genes such as runt-related transcription factor 3 (RUNX3) occurs significantly earlier in smokers than in nonsmokers with bladder cancer, and such methylation naturally increases with age [28,30]. Moreover, H19 was observed to be downregulated in immortalized human urothelial cells with long-term treatment with cigarette smoke extract (CSE), and long-term CSE treatment induced hypomethylation in the IGF2-H19 locus [28]. Compared with these results, in the present study, no significant association was observed between tobacco consumption and UCC risk (*p* = 0.071, Table 1). However, a statistically significant association was observed between age and UCC (*p* < 0.001, Table 1). This result suggested that the role of aging is more dominant than that of tobacco consumption for UCC carcinogenesis among our study participants. DNA methylation increases with age, which may play a more crucial role in UCC development [31,32,33].

We further analyzed the genotype distributions of *H19* gene polymorphisms in 431 controls and in 431 patients with UCC. We found no significant association among H19 SNPs between controls and patients with UCC in our study (Table 2). This result suggested that the direct impact of H19 SNPs on UCC carcinogenesis might be limited in the Taiwanese population. Another study focused on polymorphisms in the *H19* gene and the risk of bladder cancer in 177 patients with bladder cancer in the Netherlands. The results of that study demonstrated that patients with bladder cancer carrying the H19 rs2839698 TC genotype, but not with the homozygous CC genotype, showed a significantly decreased risk of bladder cancer [7]. Other studies examining lncRNA H19 polymorphisms and the risk of bladder cancer in the Chinese population have suggested the H19 that rs217727 polymorphism is associated with an elevated risk of bladder cancer [6,25]. However, a systematic review of studies on the association between lncRNA SNPs and the overall risk of cancer suggested that the lncRNA *H19* rs2839698 C/T and rs3024270 G/C polymorphisms, but not rs217727 C/T, are associated with overall cancer risk, including bladder cancer [34]. Compared with these results, in our study, none of the H19 SNPs rs2839898, rs3024270, and rs217727 polymorphisms was associated with the risk of UCC (Table 2). Bladder cancer has been suggested to be a heterogeneous disorder with both genetic susceptibilities and environmental exposure as risk factors [33,35,36,37]. Therefore, the regulation and influence of *H19* gene functions may depend on ethnicity, genetic susceptibility, or other epigenetic factors, which may have ultimately led to this discrepancy.

In the present study, we analyzed the distribution frequency of the clinical status and H19 SNPs. Although no significant association existed between the genotype distributions of *H19* gene polymorphisms and UCC (Table 2), we observed that in our study group of 431 UCC patients, both the H19 SNPs rs217727 and rs2107425 polymorphic variants were associated with a higher risk of muscle invasive tumors (pT2–pT4) (*p* = 0.030, Table 3; *p* = 0.025, Table 4, respectively). Most bladder cancers are urothelial carcinomas. Approximately 75% of patients with UCC have non-muscle invasive bladder cancer; 25% have muscle invasive or metastatic disease [1]. About half of non-muscle invasive bladder cancers are of a low grade [1]. However, most muscle invasive or metastatic bladder tumors are diagnosed as high grade [1,38]. Studies have suggested that the re-expressed *H19* gene in bladder cancer is associated with a higher risk of recurrent disease [7,15,16,39]. In our study, 378 of 431 UCC patients had a high histopathological grade (87.7%, Table 1), and both the H19 SNPs rs217727 and rs2107425 polymorphic variants were associated with a higher risk of muscle invasive tumors (pT2–pT4) (Table 3 and Table 4). These polymorphic variants may play a role in metastasis and are possibly linked to *H19* gene re-expression for bladder cancer progression and recurrence. rs217727 C/T is located in the exons in all transcripts [34]. Polymorphisms in the transcribed region have great potential to alter gene structure and to affect gene function. A possible mechanism to explain the discrepancy regarding the association between H19 SNPs and UCC development in different ethnicities is that microRNAs (miRNAs) may directly modify and mediate the regulation of lncRNA SNPs [34,40,41]. For example, although the miRNAs that bind to rs217727 are unidentified and have yet to be discovered [34,42], SNPs may provide a possible explanation for the alteration of the interactions between miRNAs and lncRNAs [34,43]. Moreover, Chinese patients with invasive bladder cancer carrying the H19 rs3024270 CC genotype may exhibit a decreased risk and good prognosis for bladder cancer [6]. By contrast, no significant association between the H19 rs3024270 polymorphism and muscle invasive tumors was observed in our study (data not shown). Although the expression of H19 SNPs and their correlations to clinical outcomes of bladder cancer are inconsistent among different ethnicities [6,7,25], the H19 polymorphic variants rs217727 and rs2107425 are certainly involved in UCC progression and bladder cancer regulation and may be applied as a predictor or therapeutic target for UCC. Additional well-designed studies are required to elucidate the detailed molecular mechanisms of these H19 SNPs, especially considering their interactions with miRNAs and the controversial role of lncRNA H19 as an oncogenic or tumor-suppressing regulator in UCC regulation and muscle invasive disease progression.

## 5. Conclusions

In conclusion, our study demonstrated that the H19 rs217727 CT + TT and rs2107425 CT + TT polymorphic variants are associated with a high risk of muscle invasive tumors in Taiwanese patients with UCC. H19 polymorphisms may be applied as a marker or predictor to evaluate UCC progression and prognosis.

## Figures and Tables

**Table 1:** The distributions of demographical characteristics in 431 controls and 431 patients with urothelial cell carcinoma (UCC).

Variable	Controls (N = 431) n (%)	Patients (N = 431) n (%)	*p* Value
Age (yrs)			<0.001
≤65	339 (78.7%)	166 (38.5%)	
>65	92 (21.3%)	265 (61.5%)	
Mean ± S.D.	59.4 ± 7.1	68.6 ± 11.8	<0.001
Gender			
Female	159 (36.9%)	159 (36.9%)	1.000
Male	272 (63.1%)	272 (63.1%)	
Tobacco consumption			0.071
No	275 (63.8%)	300 (69.6%)	
Yes	156 (36.2%)	131 (30.4%)	
Stage			
Non muscle invasive tumor (pTa–pT1)		235 (54.5%)	
Muscle invasive tumor (pT2–pT4)		196 (45.5%)	
Tumor T status			
Ta		90 (20.9%)	
T1-T4		341 (79.1%)	
Lymph node status			
N0		380 (88.2%)	
N1+N2		51 (11.8%)	
Metastasis			
M0		417 (96.8%)	
M1		14 (3.2%)	
Histopathologic grading			
Low grade		53 (12.3%)	
High grade		378 (87.7%)	

Student’s t test or chi-squared test was used between controls and patients with UCC.

**Table 2:** Genotype distributions of H19 gene polymorphisms in 431 controls and 431 patients with UCC.

Variable	Controls (N = 431) n (%)	Patients (N = 431) n (%)	OR (95% CI)	AOR (95% CI)
**rs2177727**				
CC	191 (44.3%)	185 (42.9%)	1.000 (reference)	1.000 (reference)
CT	188 (43.6%)	202 (46.9%)	1.109 (0.836–1.473)	1.072 (0.784–1.467)
TT	52 (12.1%)	44 (10.2%)	0.874 (0.557–1.369)	0.758 (0.458–1.255)
CT+TT	240 (55.7%)	246 (57.1%)	1.058 (0.808–1.385)	1.002 (0.744–1.350)
**rs2107425**				
CC	171 (39.7%)	152 (35.3%)	1.000 (reference)	1.000 (reference)
CT	190 (44.1%)	213 (49.4%)	1.261 (0.941–1.691)	1.245 (0.901–1.721)
TT	70 (16.2%)	66 (15.3%)	1.061 (0.710–1.584)	0.978 (0.625–1.529)
CT+TT	260 (60.3%)	279 (64.7%)	1.207 (0.916–1.591)	1.172 (0.865–1.589)
**rs2839698**				
CC	192 (44.5%)	206 (47.8%)	1.000 (reference)	1.000 (reference)
CT	184 (42.7%)	170 (39.4%)	0.861 (0.647–1.147)	0.907 (0.660–1.247)
TT	55 (12.8%)	55 (12.8%)	0.932 (0.611–1.422)	0.881 (0.555–1.399)
CT+TT	239 (55.5%)	225 (52.2%)	0.877 (0.671–1.147)	0.901 (0.669–1.212)
**rs3024270**				
CC	120 (27.8%)	114 (26.5%)	1.000 (reference)	1.000 (reference)
GC	208 (48.3%)	210 (48.7%)	1.063 (0.772–1.464)	1.316 (0.920–1.884)
GG	103 (23.9%)	107 (24.8%)	1.094 (0.753–1.587)	1.189 (0.786–1.797)
GC+GG	311 (72.2%)	317 (73.5%)	1.073 (0.795–1.449)	1.271 (0.909–1.778)
**rs3741219**				
AA	185 (42.9%)	192 (44.5%)	1.000 (reference)	1.000 (reference)
GA	190 (44.1%)	181 (42%)	0.918 (0.689–1.223)	0.978 (0.711–1.345)
GG	56 (13%)	58 (13.5%)	0.998 (0.656–1.517)	0.912 (0.576–1.444)
GA+GG	246 (57.1%)	239 (55.5%)	0.936 (0.715–1.225)	0.962 (0.713–1.296)

Bold font indicates statistical significance (*p* < 0.05). The odds ratio (OR) with their 95% confidence intervals (CI) were estimated by logistic regression models. The adjusted odds ratio (AOR) with their 95% CI were estimated by multiple logistic regression models after controlling for age, sex, and tobacco consumption.

**Table 3:** Distribution frequency of the clinical status and H19 rs2177727 genotype frequencies in 431 UCC patients.

	H19 (rs2177727)			
Variable	CC (%) (n = 185)	CT+TT (%) (n = 246)	OR (95% CI)	*p* Value
**Stage**				
Non muscle invasive tumor (pTa–pT1)	112 (60.5%)	123 (50%)	1.000 (reference)	
Muscle invasive tumor (pT2–pT4)	73 (39.5%)	123 (50%)	**1.534 (1.042–2.258)**	**0.030**
**Tumor T status**				
Ta	41 (22.2%)	49 (19.9%)	1.000 (reference)	
T1–T4	144 (77.8%)	197 (80.1%)	1.145 (0.717–1.826)	0.571
**Lymph node status**				
N0	165 (89.2%)	215 (87.4%)	1.000 (reference)	
N1+N2	20 (10.8%)	31 (12.6%)	1.190 (0.654–2.162)	0.569
**Metastasis**				
M0	179 (96.8%)	238 (96.7%)	1.000 (reference)	
M1	6 (3.2%)	8 (3.3%)	1.003 (0.342–2.941)	0.996
**Histopathologic grading**				
Low grade	22 (11.9%)	31 (12.6%)	1.000 (reference)	
High grade	163 (88.1%)	215 (87.4%)	0.936 (0.523–1.677)	0.824

Bold font indicates statistical significance (*p* < 0.05). The OR with their 95% CI were estimated by logistic regression models.

**Table 4:** Distribution frequency of the clinical status and H19 rs2107425 genotype frequencies in 431 UCC patients.

	H19 (rs2107425)			
Variable	CC (%) (n = 152)	CT+TT (%) (n = 279)	OR (95% CI)	*p* Value
**Stage**				
Non muscle invasive tumor (pTa–pT1)	94 (61.8%)	141 (50.5%)	1.000 (reference)	
Muscle invasive tumor (pT2–pT4)	58 (38.2%)	138 (49.5%)	**1.586 (1.06–2.373)**	**0.025**
**Tumor T status**				
Ta	37 (24.3%)	53 (19%)	1.000 (reference)	
T1–T4	115 (75.7%)	226 (81%)	1.372 (0.852–2.209)	0.193
**Lymph node status**				
N0	136 (89.5%)	244 (87.5%)	1.000 (reference)	
N1+N2	16 (10.5%)	35 (12.5%)	1.219 (0.651–2.284)	0.536
**Metastasis**				
M0	150 (98.7%)	267 (95.7%)	1.000 (reference)	
M1	2 (1.3%)	12 (4.3%)	3.371 (0.744–15.262)	0.115
**Histopathologic grading**				
Low grade	16 (10.5%)	37 (13.3%)	1.000 (reference)	
High grade	136 (89.5%)	242 (86.7%)	0.769 (0.413–1.435)	0.410

Bold font indicates statistical significance (*p* < 0.05). The OR with their 95% CI were estimated by logistic regression models.

**Table 5:** Risk of disease-specific mortality on genotype distributions of H19 gene polymorphisms among 264 UCC patients over 65 years old.

Variable	N	Number of Disease-Specific Mortality	HR (95% CI)	AHR (95% CI)
**rs2177727**				
CC	112	17	1.000 (reference)	1.000 (reference)
CT+TT	152	30	1.308 (0.721–2.373)	1.404 (0.770–2.561)
**rs2107425**				
CC	93	11	1.000 (reference)	1.000 (reference)
CT+TT	171	36	1.862 (0.948–3.659)	**2.043 (1.029–4.059)**
**rs2839698**				
CC	128	27	1.000 (reference)	1.000 (reference)
CT+TT	136	20	0.669 (0.375–1.195)	0.668 (0.374–1.194)
**rs3024270**				
CC	75	14	1.000 (reference)	1.000 (reference)
GC+GG	189	33	0.934 (0.499–1.749)	0.892 (0.470–1.691)
**rs3741219**				
AA	120	25	1.000 (reference)	1.000 (reference)
GA+GG	144	22	0.678 (0.382–1.204)	0.683 (0.383–1.217)

Bold font indicates statistical significance (*p* < 0.05). The hazrds ratio (HR) with their 95% CI were estimated by Cox proportional hazards model. The adjusted hard ratio (AHR) with their 95% CI were estimated by multiple Cox proportional hazards model after controlling for age, sex, and tobacco consumption.

## References

[1] Kamat A.M., Hahn N.M., Efstathiou J.A., Lerner S.P., Malmstrom P.U., Choi W., Guo C.C., Lotan Y., Kassouf W. (2016). Bladder cancer. Lancet.

[2] Tung M.C., Wen Y.C., Wang S.S., Lin Y.W., Chow J.M., Yang S.F., Chien M.H. (2019). Impact of long non-coding rna hotair genetic variants on the susceptibility and clinicopathologic characteristics of patients with urothelial cell carcinoma. J. Clin. Med..

[3] Hung C.F., Yang C.K., Ou Y.C. (2016). Urologic cancer in taiwan. Jpn. J. Clin. Oncol..

[4] Burger M., Catto J.W., Dalbagni G., Grossman H.B., Herr H., Karakiewicz P., Kassouf W., Kiemeney L.A., La Vecchia C., Shariat S. (2013). Epidemiology and risk factors of urothelial bladder cancer. Eur. Urol..

[5] Chang C.H., Liu C.S., Liu H.J., Huang C.P., Huang C.Y., Hsu H.T., Liou S.H., Chung C.J. (2016). Association between levels of urinary heavy metals and increased risk of urothelial carcinoma. Int. J. Urol..

[6] Li Z., Niu Y. (2018). Association between lncrna h19 (rs217727, rs2735971 and rs3024270) polymorphisms and the risk of bladder cancer in chinese population. Minerva Urologica e Nefrologica = Ital. J. Urol. Nephrol..

[7] Verhaegh G.W., Verkleij L., Vermeulen S.H., den Heijer M., Witjes J.A., Kiemeney L.A. (2008). Polymorphisms in the h19 gene and the risk of bladder cancer. Eur. Urol..

[8] Droop J., Szarvas T., Schulz W.A., Niedworok C., Niegisch G., Scheckenbach K., Hoffmann M.J. (2017). Diagnostic and prognostic value of long noncoding rnas as biomarkers in urothelial carcinoma. PLoS ONE.

[9] Wang J., Yang K., Yuan W., Gao Z. (2018). Determination of serum exosomal h19 as a noninvasive biomarker for bladder cancer diagnosis and prognosis. Med. Sci. Monit. Int. Med. J. Exp. Clin. Res..

[10] Yin Z., Cui Z., Li H., Li J., Zhou B. (2018). Polymorphisms in the h19 gene and the risk of lung cancer among female never smokers in shenyang, china. BMC Cancer.

[11] Poirier F., Chan C.T., Timmons P.M., Robertson E.J., Evans M.J., Rigby P.W. (1991). The murine h19 gene is activated during embryonic stem cell differentiation in vitro and at the time of implantation in the developing embryo. Development.

[12] Zhang Y., Tycko B. (1992). Monoallelic expression of the human h19 gene. Nat. Genet..

[13] Ariel I., Lustig O., Schneider T., Pizov G., Sappir M., De-Groot N., Hochberg A. (1995). The imprinted h19 gene as a tumor marker in bladder carcinoma. Urology.

[14] Ariel I., Ayesh S., Perlman E.J., Pizov G., Tanos V., Schneider T., Erdmann V.A., Podeh D., Komitowski D., Quasem A.S. (1997). The product of the imprinted h19 gene is an oncofetal rna. Mol. Pathol..

[15] Cooper M.J., Fischer M., Komitowski D., Shevelev A., Schulze E., Ariel I., Tykocinski M.L., Miron S., Ilan J., de Groot N. (1996). Developmentally imprinted genes as markers for bladder tumor progression. J. Urol..

[16] Elkin M., Shevelev A., Schulze E., Tykocinsky M., Cooper M., Ariel I., Pode D., Kopf E., de Groot N., Hochberg A. (1995). The expression of the imprinted h19 and igf-2 genes in human bladder carcinoma. FEBS Lett..

[17] Zhu Z., Xu L., Wan Y., Zhou J., Fu D., Chao H., Bao K., Zeng T. (2018). Inhibition of e-cadherin expression by lnc-rna h19 to facilitate bladder cancer metastasis. Cancer Biomark. Sect. A Dis. Mark..

[18] Cui P., Zhao Y., Chu X., He N., Zheng H., Han J., Song F., Chen K. (2018). Snp rs2071095 in lincrna h19 is associated with breast cancer risk. Breast Cancer Res. Treat..

[19] Lin Y., Fu F., Chen Y., Qiu W., Lin S., Yang P., Huang M., Wang C. (2017). Genetic variants in long noncoding rna h19 contribute to the risk of breast cancer in a southeast china han population. OncoTargets Ther..

[20] Abdollahzadeh S., Ghorbian S. (2018). Association of the study between lncrna-h19 gene polymorphisms with the risk of breast cancer. J. Clin. Lab. Anal..

[21] Guo Q.Y., Wang H., Wang Y. (2017). Lncrna h19 polymorphisms associated with the risk of oscc in chinese population. Eur. Rev. Med. Pharmacol. Sci..

[22] Gong W.J., Yin J.Y., Li X.P., Fang C., Xiao D., Zhang W., Zhou H.H., Li X., Liu Z.Q. (2016). Association of well-characterized lung cancer lncrna polymorphisms with lung cancer susceptibility and platinum-based chemotherapy response. Tumour Biol..

[23] Li L., Guo G., Zhang H., Zhou B., Bai L., Chen H., Zhao Y., Yan Y. (2018). Association between h19 snp rs217727 and lung cancer risk in a chinese population: A case control study. BMC Med. Genet..

[24] Yang M.L., Huang Z., Wang Q., Chen H.H., Ma S.N., Wu R., Cai W.S. (2018). The association of polymorphisms in lncrna-h19 with hepatocellular cancer risk and prognosis. Biosci. Rep..

[25] Hua Q., Lv X., Gu X., Chen Y., Chu H., Du M., Gong W., Wang M., Zhang Z. (2016). Genetic variants in lncrna h19 are associated with the risk of bladder cancer in a chinese population. Mutagenesis.

[26] Cheng L., Montironi R., Davidson D.D., Lopez-Beltran A. (2009). Staging and reporting of urothelial carcinoma of the urinary bladder. Mod. Pathol..

[27] Hung S.C., Wang S.S., Yang C.K., Li J.R., Cheng C.L., Ou Y.C., Ho H.C., Chiu K.Y., Chen C.S. (2017). Comparison of efficacy of adjuvant mec (methotrexate, epirubicin and cisplatin) and gc (gemcitabine and cisplatin) in advanced upper tract urothelial carcinoma. Anticancer Res..

[28] Chen L.M., Nergard J.C., Ni L., Rosser C.J., Chai K.X. (2013). Long-term exposure to cigarette smoke extract induces hypomethylation at the runx3 and igf2-h19 loci in immortalized human urothelial cells. PLoS ONE.

[29] Marsit C.J., Karagas M.R., Danaee H., Liu M., Andrew A., Schned A., Nelson H.H., Kelsey K.T. (2006). Carcinogen exposure and gene promoter hypermethylation in bladder cancer. Carcinogenesis.

[30] Wolff E.M., Liang G., Cortez C.C., Tsai Y.C., Castelao J.E., Cortessis V.K., Tsao-Wei D.D., Groshen S., Jones P.A. (2008). Runx3 methylation reveals that bladder tumors are older in patients with a history of smoking. Cancer Res..

[31] Schulz W.A., Goering W. (2016). DNA methylation in urothelial carcinoma. Epigenomics.

[32] Volanis D., Papadopoulos G., Doumas K., Gkialas I., Delakas D. (2011). Molecular mechanisms in urinary bladder carcinogenesis. J. B.U.ON. Off. J. Balkan Union Oncol..

[33] Byun H.M., Wong H.L., Birnstein E.A., Wolff E.M., Liang G., Yang A.S. (2007). Examination of igf2 and h19 loss of imprinting in bladder cancer. Cancer Res..

[34] Lv Z., Xu Q., Yuan Y. (2017). A systematic review and meta-analysis of the association between long non-coding rna polymorphisms and cancer risk. Mutat. Res..

[35] Stewart S.L., Cardinez C.J., Richardson L.C., Norman L., Kaufmann R., Pechacek T.F., Thompson T.D., Weir H.K., Sabatino S.A. (2008). Surveillance for Cancers Associated with Tobacco Use—United States, 1999–2004.

[36] Casadevall D., Kilian A.Y., Bellmunt J. (2017). The prognostic role of epigenetic dysregulation in bladder cancer: A systematic review. Cancer Treat. Rev..

[37] Kim W.J., Bae S.C. (2008). Molecular biomarkers in urothelial bladder cancer. Cancer Sci..

[38] Moch H., Cubilla A.L., Humphrey P.A., Reuter V.E., Ulbright T.M. (2016). The 2016 who classification of tumours of the urinary system and male genital organs—Part A: Renal, penile, and testicular tumours. Eur. Urol..

[39] Ariel I., Sughayer M., Fellig Y., Pizov G., Ayesh S., Podeh D., Libdeh B.A., Levy C., Birman T., Tykocinski M.L. (2000). The imprinted h19 gene is a marker of early recurrence in human bladder carcinoma. Mol. Pathol..

[40] Kallen A.N., Zhou X.B., Xu J., Qiao C., Ma J., Yan L., Lu L., Liu C., Yi J.S., Zhang H. (2013). The imprinted h19 lncrna antagonizes let-7 micrornas. Mol. Cell.

[41] Moran V.A., Perera R.J., Khalil A.M. (2012). Emerging functional and mechanistic paradigms of mammalian long non-coding rnas. Nucleic Acids Res..

[42] Yang C., Tang R., Ma X., Wang Y., Luo D., Xu Z., Zhu Y., Yang L. (2015). Tag snps in long non-coding rna h19 contribute to susceptibility to gastric cancer in the chinese han population. Oncotarget.

[43] Wu H., Zheng J., Deng J., Hu M., You Y., Li N., Li W., Lu J., Zhou Y. (2013). A genetic polymorphism in lincrna-uc003opf.1 is associated with susceptibility to esophageal squamous cell carcinoma in chinese populations. Carcinogenesis.

